# Complete genome sequence of a novel nege-like virus in aphids (genus *Indomegoura*)

**DOI:** 10.1186/s12985-021-01552-w

**Published:** 2021-04-13

**Authors:** Yu-Hua Qi, Liao-Yuan Xu, Jing Zhai, Zhuang-Xin Ye, Gang Lu, Jian-Ping Chen, Chuan-Xi Zhang, Jun-Min Li

**Affiliations:** 1 College of Plant Protection, Fujian Agriculture and Forestry University, Fuzhou, 350002 China; 2 State Key Laboratory for Managing Biotic and Chemical Threats To the Quality and Safety of Agro-Products, Key Laboratory of Biotechnology in Plant Protection of Ministry of Agriculture and Zhejiang Province, Institute of Plant Virology, Ningbo University, Ningbo, 315211 China; 3 Popularization of Agricultural Technical Station of Ningbo, Ningbo, 315012 China

**Keywords:** Metagenomic sequencing, Small interfering RNA, Virus discovery, Insect specific virus

## Abstract

**Background:**

Aphids are important vectors of numerous plant viruses. Besides plant viruses, a number of insect specific viruses (ISVs), such as nege/nege-like viruses, have been recently discovered in aphids of the genera *Aphis*, *Rhopalosiphum*, and *Sitobion*.

**Findings:**

In this study, the complete genome sequence of a novel nege-like virus, tentatively named “Indomegoura nege-like virus 1” (INLV1), was identified in aphids of the genus *Indomegoura*. INLV1 possessed a single positive-stranded RNA genome with 8945 nucleotides, which was predicted to contain three typical open reading frames (ORFs) of negeviruses (including ORF1, ORF2, and ORF3), a 44-nt 5′ untranslated region (UTR) and a 98-nt 3′ UTR. Five conserved domains were predicted for INLV1, including an Alphavirus-like methyltransferase domain, a RNA virus helicase core domain, and a RNA-dependent RNA polymerase domain (RdRP) in ORF1, a DISB-ORF2_chro domain in ORF2, and a SP24 domain in ORF3. According to the maximum likelihood phylogenetic tree based on RdRP, INLV1 was grouped with barley aphid RNA virus 1 and Hubei virga-like virus 4, together with another two invertebrate viruses, which formed a distinct clade in the proposed group Centivirus. The alignment of RdRP domains for INLV1 and other nege/kita-like viruses suggested that RdRP of INLV1 contained the permuted C (GDD)- A [DX(4–5)D] –B [GX(2–3)TX(3)N] motifs, which were conserved in the Centivirus and Sandewavirus groups. Furthermore, the high abundance and typical characteristics of INLV1 derived small interfering RNAs clearly showed the active replication of INLV1 in the aphid *Indomegoura*.

**Conclusion:**

INLV1 is the first nege-like virus infecting aphids of the genus *Indomegoura*. As far as we know, it is also the first ISV revealed in this aphid genus.

**Supplementary Information:**

The online version contains supplementary material available at 10.1186/s12985-021-01552-w.

## Background

Aphids, which belong to the order Hemiptera, family *Aphididae*, are one of the most serious pests of agricultural and horticultural crops. Aphids will cause damage through direct feeding or by means of vectors of many important plant viruses [1]. Apart from plant viruses, recent studies have indicated that aphids are known to harbor numerous novel insect specific viruses (ISVs) belonging to the families *Dicistroviridae* and *Iflaviridae*, and others from various unclassified taxa [2–7]. Negeviruses are a newly proposed group of ISVs well-known for their wide geographic distribution and broad host range [8–10]. The genome of negeviruses is a single positive-sense RNA with the size of 9–10 kb, which encodes three open reading frames (ORFs). Negeviruses are currently classified into two distinct phylogenetic clades called Nelorpivirus and Sandewavirus, and they are also closely related to plant viruses in the family *Kitaviridae* [11–13].

A few nege/nege-like viruses have hitherto been reported in aphids. The first aphid nege-like virus was discovered in soybean aphid (*Aphis glycines*), which was obtained from Ohio State University using Next Generation Sequencing [14]. More recently, a number of nege/kita-like viruses have been discovered in the other two genera of aphids (*Rhopalosiphum* and *Sitobion*). According to phylogenetic analysis, the nege/kita-like viruses infecting these two aphid genera can be classified into two new distinct clades, tentatively designated as Centivirus and Aphiglyvirus, respectively [13].

## Main text

In this study, a novel nege-like virus was discovered in aphids of the genus *Indomegoura*. The aphids were harvested from the host plant *Hemerocallis fulva* at Ningbo University, Ningbo, China in 2020. Then, we used TRIzol reagent (Invitrogen, MA, USA) to extract total RNA from a pool of ten aphids. The Nano Drop spectrophotometer (Thermo Scientific, MA, USA) was used to determine the RNA content. Paired-end (150 bp) sequencing of the RNA library was performed using the Illumina HiSeq 4000 sequencer (Novogene, Tianjin, China). Afterwards, the 22,045,205 pairs of raw reads generated were subjected to quality trimming and de novo assembly by adopting Trinity (version 2.8.5) with the default parameters [15]. To determine the accurate aphid species, all the 63,158 assembled contigs were compared with cytochrome oxidase subunit 1 (COI) records derived from the Barcode of Life Data (BOLD) Systems (http://www.boldsystems.org/), and later the potential aphid COI sequence was extracted. The aphid COI sequence was then compared with the nucleotide (nt) database in NCBI, which showed high homology (97% similarities) to the COI of *Indomegoura indica* (Accession number: NC_045897.1), confidentially indicating that the collected aphid species were highly similar to *I. indica* and belonged to the genus *Indomegoura*. The COI sequence of aphids *Indomegoura* was further confirmed by Sanger sequencing and stored in GenBank under the accession number MW533423 (Additional file 1: File S1).

To identify the potential viral-like contigs in the transcriptome, the assembled contigs were searched against the local generated virus database with the sequences retrieved from NCBI viral reference database (https://www.ncbi.nlm.nih.gov/genome/viruses). As a result, a confidently nege-like viral contig was discovered in aphids, which represented almost the complete viral genome with the length of 8876 nt. To investigate the transcript abundance and coverage of the contig, the adaptor- and quality-trimmed reads from the transcriptome were mapped back to this contig using Bowtie2 and Samtools. As a result, high coverage (290X) was confirmed for this nege-like viral contig. Thereafter, the identified viral contig was further compared with the entire NCBI nucleotide (NT) and non-redundant (NR) protein database to avoid false positive results (Additional file 3: Table S1). Then, the viral contig was confirmed with reverse transcription-PCR (RT-PCR), followed by Sanger sequencing. Furthermore, the full genome of the nege-like virus was successfully achieved by the rapid amplification of cDNA ends (RACE) with SMARTer® RACE 5′/3′ kit (Takara, Dalian, China). The primers used for RT-PCR and RACE are listed in Additional file 4: Table S2. The novel nege-like virus from aphids of genus *Indomegoura* was temporarily named “Indomegoura nege-like virus 1” (INLV1), and its full genome sequence was deposited in GenBank with the accession number MW285725 (Additional file 2: File S2).

**Fig. 1:**
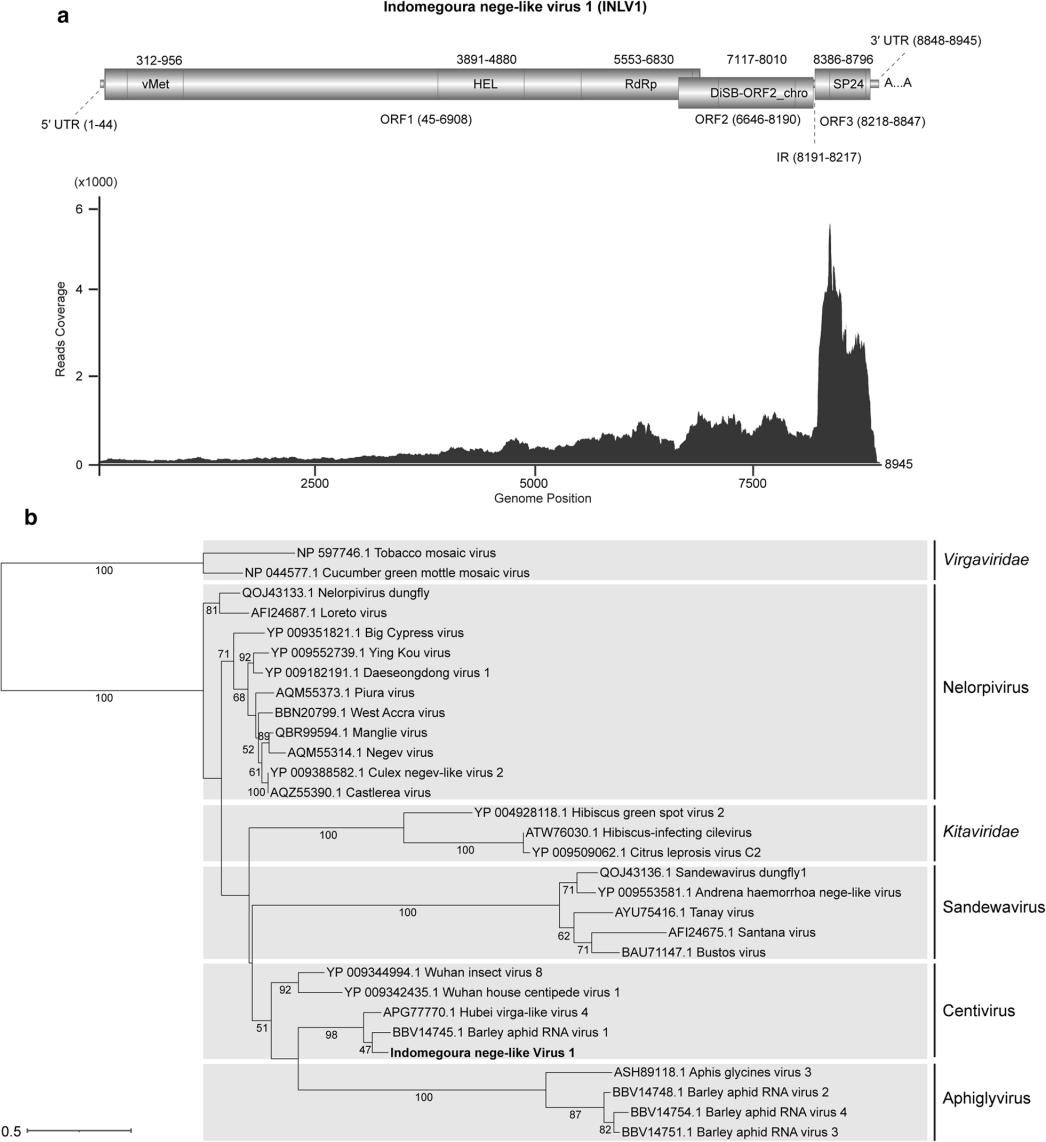
**a** Genome organization and transcriptome raw read coverage of Indomegoura nege-like virus 1 (INLV1). vMet, Alphavirus-like methyltransferase domain; HEL, RNA virus helicase core domain; RdRp, RNA-dependent RNA polymerase domain; UTR, untranslated region; IR, intergenic region. **b** Maximum likelihood phylogenetic tree based on the RdRp domain of INLV1, previously reported representative nege/nege-like viruses, and plant viruses in the families *Kitaviridae* and *Virgaviridae*

The RT-PCR and Sanger sequencing results confirmed the sequences of the assembled viral-like contig (with a few corrections of the nucleotides). Furthermore, the complete 5′ and 3′ untranslated region (UTR) were obtained using RACE technology followed by Sanger sequencing, and the full genome sequences of INLV1 was successfully reconstructed. INLV1 had a genome size of 8945 nt (excluding polyA), which was the most homologous to Hubei virga-like virus 4 (HVLV-4) (accession number APG77770.1) and barley aphid RNA virus 1 (BARV-1) (accession number BBV14745.1), with the amino acid (aa) sequence identities of 59.00% and 58.47%, respectively. In terms of the genome organization, INLV1 contained three typical negevirus ORFs (ORF1, ORF2, and ORF3) predicted using the Expasy online server (https://web.expasy.org/translate/), a 44-nt 5′ UTR and a 98-nt 3′ UTR (nucleotide position in the genome: 8848–8945 nt) (Fig. 1a). Additionally, the conserved domains predicted using InterProScan (https://www.ebi.ac.uk/interpro) suggested that the long ORF1 (nucleotide position in the genome: 45-6908 nt) consisted of an Alphavirus-like methyltransferase domain (vMet, IPR002588), a RNA virus helicase core domain (HEL, PF01443), and a RNA-dependent RNA polymerase domain (RdRP, PF00978). In addition, RNA ribosomal methyltransferase domain (FstJ), which was demonstrated to be present or absent in various negeviruses [10, 12, 13], was not detected in the ORF1 of INLV1, indicating that FstJ might not be well-conserved in the taxon Negevirus. ORF2 and ORF3 of INLV1 possessed the conserved domains of DiSB-ORF2_chro (a putative virion glycoprotein, PF16506) and SP24 (a putative virion membrane protein, PF16504), respectively (Fig. 1a), which were similar to another negevirus isolated from *Aedes vexans* mosquitoes in Finland [16]. According to previous studies, overlaps between different ORFs of negeviruses are common [8, 10]. In our study, an overlap between ORF1 and ORF2 by 263-nt was also found with different frames in INLV1 (Fig. 1a). To further understand the abundance and coverage of sequenced reads derived from INLV1, we realigned the RNA-seq reads to the confirmed full genome of INLV1. Noteworthily, viral reads were apparently accumulated within the 3′ region of the genome, especially in ORF3 (Fig. 1a), consistent with the recently reported negeviruses discovered in a dungfly [10]. In addition, the transmembrane domains of INLV1 ORF3 were predicted by the TMHMM server v. 2.0 (http://www.cbs.dtu.dk/services/TMHMM/). As a result, the four transmembrane domains were evidently present in the ORF3 of INLV1 (Additional file 6: Figure S1), indicating that SP24 was probably an integral membrane protein of INLV1, conforming to previous report [17].

To further evaluate the taxonomical status of INLV1, we aligned the conserved RdRP domain of INLV1 and the previously reported nege/nege-like viruses by MAFFT (version 7.450), and further trimmed the gaps by Gblock [18]. Besides, the substitution model was evaluated by ModelTest-NG, and a maximum likelihood (ML) tree was constructed using IQ-tree with 1000 bootstrap replications [19, 20]. Two plant viruses in the family *Virgaviridae*, Tobacco mosaic virus (NP_597746.1) and Cucumber green mottle mosaic virus (NP_044577.1), were used as outgroup. According to recent phylogenetic study on aphid nege/kita-like viruses, it is proposed that the two newly identified groups, Centivirus and Aphiglyvirus, together with the Negevirus subgroups (Nelorpivirus and Sandewavirus), can be classified into a novel viral family or assigned to the family *Kitaviridae* [13]. In this study, the reconstructed phylogenetic ML tree based on the viral RdRP domain sequences indicated that INLV1 was clearly grouped with BARV-1 and HVLV-4, together with another two invertebrate viruses, which formed a distinct group in the clade Centivirus closely related to Aphiglyvirus (Fig. 1b).Using MegAlign (version 7.1.0) and BioEdit Sequence Alignment Editor (version 7.1.11) [21], we aligned INLV1 and the related viruses based on the predicted RdRP protein/nucleotide sequences of INLV1 and the previously reported nege/kita-like viruses, so as to determine the homology of INLV1 with the related viruses. Previous study indicates that the RdRP of nege/kita-like viruses contains three conserved motifs, namely, motif A [DX(4–5)D], motif B [GX(2–3)TX(3)N], and motif C (GDD), in the canonical order A-B-C or the permuted order C-A-B [22]. In our study, these two motif types of nege/kita-like viruses were also observed, and the RdRP domain of INLV1 showed the clear permuted C-A-B motif pattern (Additional file 7: Figure S2). More interestingly, the canonical A-B-C type of RdRPs exclusively belonged to the groups Nelorpivirus and Aphiglyvirus, as well as the plant virus of families *Kitaviridae* and *Virgaviridae*, whereas the permuted C-A-B pattern was observed in the groups Centivirus and Sandewavirus (Additional file 7: Figure S2), consistent with the taxonomical status of each group in the phylogenic tree (Fig. 1b). Furthermore, we compared the aa/nt identity of INLV1 RdRP sequences with other reported nege/kita-like viruses. As a result, INLV1 was the most closely related to BARV-1 and HVLV-4 in the group Centivirus, with the aa (nt) identities of 77.2% (69.2%) and 76.3% (69.0%), respectively (Table 1). For the phylogenetically related aphiglyviruses (Fig. 1b), INLV1 shared 31.4–32.9% (aa) and 47.5–50.3% (nt) identities (Table 1).

**Table 1:** Amino acid/nucleotide identity values based on the conserved amino acid sequence and nucleotide sequence of the RdRp domain

	Identity	1	2	3	4	5	6	7	8	9	10	11	12	13	14	15	16	17	18
Centivirus	**INLV1**	***	**77.2**	**76.3**	**50.5**	**47.0**	**46.1**	**46.9**	**46.0**	**31.4**	**32.9**	**32.9**	**31.5**	**37.4**	**32.7**	**33.6**	**32.5**	**30.4**	**30.0**
	BARV-1	69.2	***	**77.6**	**52.6**	**50.5**	**48.3**	**43.8**	**46.8**	**32.2**	**32.8**	**32.0**	**33.0**	**36.5**	**36.4**	**34.5**	**33.8**	**33.1**	**31.7**
	HVLV-4	69.0	70.8	***	**51.3**	**49.2**	**46.3**	**43.6**	**44.7**	**32.5**	**33.0**	**33.7**	**32.8**	**36.9**	**34.8**	**35.0**	**33.1**	**33.1**	**31.5**
	WHCV-1	57.2	57.2	56.6	***	**66.0**	**54.1**	**49.1**	**53.0**	**34.3**	**37.3**	**35.4**	**35.5**	**39.4**	**35.4**	**35.3**	**36.2**	**33.6**	**34.2**
	WhIV-8	53.1	54.1	54.1	62.3	***	**54.3**	**49.3**	**51.8**	**33.1**	**36.1**	**34.2**	**32.2**	**39.7**	**33.1**	**35.9**	**34.2**	**32.7**	**33.0**
Nelorpivirus	MaV	50.1	54.7	50.0	55.9	56.7	***	**61.2**	**69.1**	**28.4**	**31.5**	**29.8**	**31.6**	**35.3**	**33.1**	**35.5**	**33.3**	**32.8**	**32.6**
	LORV	53.3	50.3	51.2	54.6	55.9	61.1	***	**63.4**	**29.5**	**30.3**	**30.3**	**30.8**	**36.7**	**36.1**	**37.3**	**32.5**	**31.0**	**29.6**
	BCPV	49.5	52.3	51.2	56.0	54.4	64.2	60.7	***	**30.0**	**31.5**	**32.0**	**32.0**	**36.0**	**37.0**	**37.6**	**32.8**	**32.3**	**30.3**
Aphiglyvirus	BARV-2	50.0	46.1	47.8	48.2	45.1	43.0	45.0	43.2	***	**79.5**	**79.2**	**53.0**	**31.2**	**28.6**	**28.8**	**25.4**	**25.4**	**24.5**
	BARV-3	50.3	45.7	47.2	50.5	47.6	44.2	43.7	43.5	72.8	***	**83.1**	**55.1**	**33.0**	**29.9**	**30.1**	**26.3**	**27.1**	**26.6**
	BARV-4	49.7	46.4	48.1	48.3	46.4	43.7	43.2	45.2	73.1	74.0	***	**55.1**	**32.3**	**30.1**	**30.3**	**26.1**	**27.1**	**25.9**
	AGV-3	47.5	44.3	44.2	47.4	44.6	42.1	43.5	41.6	62.2	60.9	59.7	***	**34.6**	**34.0**	**34.2**	**26.6**	**26.3**	**26.7**
*Kitaviridae*	HGSV-2	47.7	44.7	45.3	48.1	49.9	46.1	48.0	47.1	47.0	44.7	46.4	46.4	***	**51.7**	**50.6**	**30.0**	**30.3**	**28.3**
	CiLV-C2	45.5	45.2	46.1	48.8	46.7	46.6	49.5	47.6	43.9	44.1	45.0	45.2	55.8	***	**77.1**	**27.6**	**28.1**	**25.5**
	CiLV-C	45.9	44.6	45.1	48.2	46.9	48.1	49.5	45.9	44.9	46.5	43.8	44.9	57.3	68.8	***	**29.6**	**26.6**	**24.9**
Sandewavirus	SVD1	48.7	47.6	47.3	50.3	47.6	46.9	45.8	45.4	42.0	43.7	42.1	41.4	45.1	44.4	43.5	***	**60.1**	**59.4**
	TANAV	43.8	45.3	45.7	45.4	46.3	44.5	44.0	44.1	41.2	41.7	40.9	40.9	45.2	44.2	40.8	61.7	***	**66.9**
	BUSV	45.9	43.8	46.8	46.1	46.2	44.0	44.9	44.6	41.9	41.9	41.2	39.6	43.3	43.2	40.4	62.6	63.8	***

Bold text indicates amino acid identity. Non-bold text indicates nucleotide identity. The numbers 1–18 represent viruses from INLV1-BUSV in the left column. Virus names and GenBank accessions numbers are listed in Additional file 5: Table S3

**Fig. 2:**
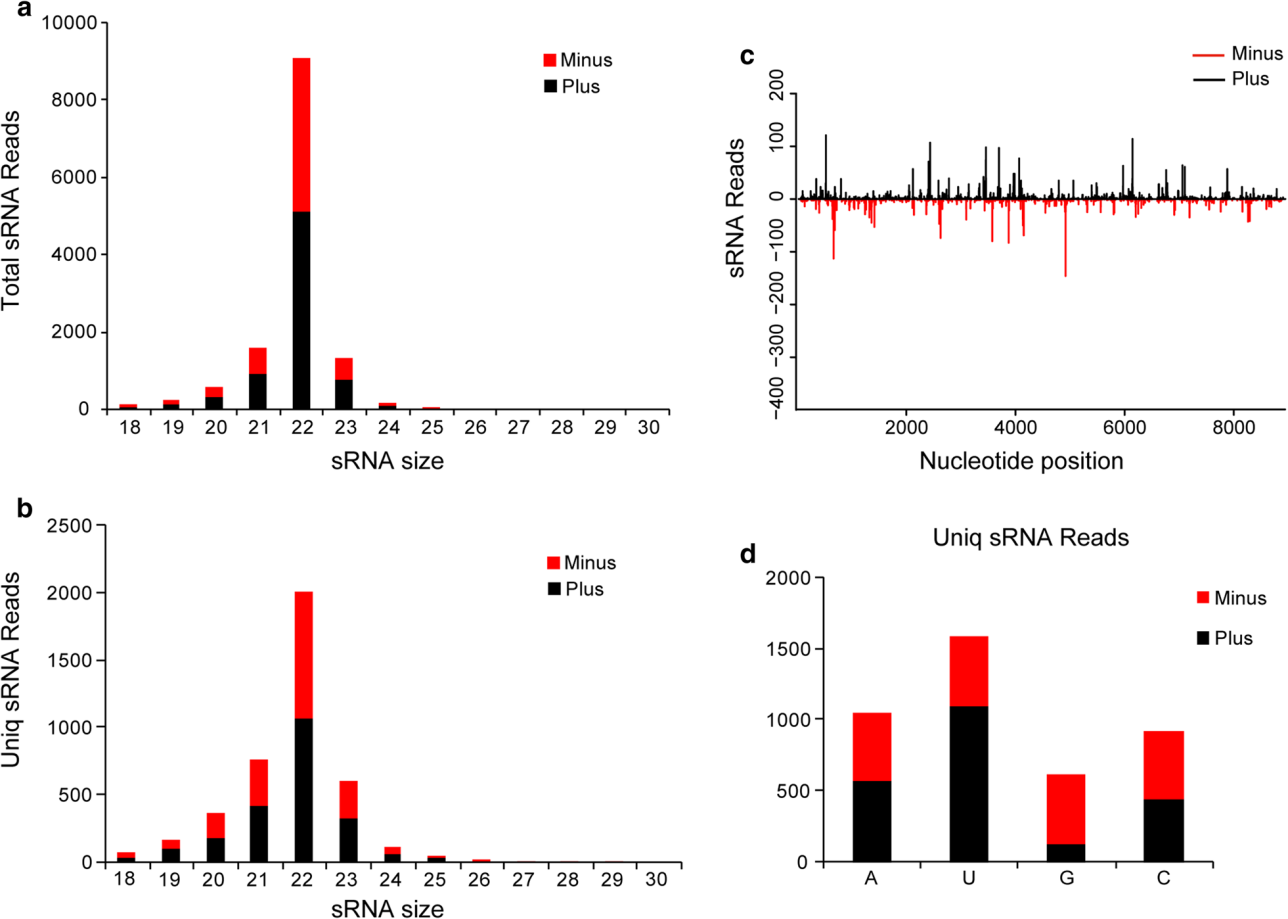
Analysis of virus derived small interfering RNAs (vsiRNAs) of INLV1. The size distribution of INLV1-derived siRNAs of total reads (**a**) or unique reads (**b**). **c** Distribution of INLV1-derived siRNA on the viral genome. **d** 5′ terminal nucleotide preference of siRNAs derived from INLV1. Black represents siRNAs derived from the sense genomic strand (Plus), and red represents small RNAs derived from the antisense genomic strand (Minus)

To explore small interfering RNA (siRNA) based anti-viral immunity in aphid host, small RNAs (sRNA) of the aphids were sequenced and virus derived siRNAs (vsiRNAs) were comprehensively characterized. In brief, a sRNA library was prepared using the Illumina TruSeq small RNA sample preparation kit (Illumina, San Diego, CA, USA), and sRNA sequencing was performed by Novogene on an Illumina HiSeq 2500 platform. The sRNA reads were pretreated (removal of adapters, low quality, and junk sequences) and sRNAs with the length of 18-nt to 30-nt were extracted. The processed sRNA reads were mapped back to the full viral genome sequence of INLV1 using Bowtie with zero mismatches. vsiRNAs were further analyzed using the custom perl scripts and the Linux bash scripts. As a result, a total of 13,203 (4,181 unique) vsiRNAs perfectly mapped to INLV1 genome were identified, accounting for 0.06% (0.48% unique) of the sRNA library. The vsiRNAs were mostly 22-nt long (accounting for 69.0% and 48.1% of total and unique vsiRNAs, respectively), and they were equally derived from the sense and antisense strands of the viral genome (Fig. 2a, b). Besides, equal distribution alongside the viral genome and a strong A/U bias in the 5′-terminal nucleotide of vsiRNAs was also observed (Fig. 2c, d), which have been characterized for vsiRNAs derived from various organisms, including insects [23]. These typical characteristics of INLV1 derived siRNAs strongly suggested that the active involvement of RNA interference antiviral pathway in the aphid genus *Indomegoura*.

## Conclusions

In addition to the recent discoveries of nege/nege-like viruses in various aphid genera including *Aphis*, *Rhopalosiphum*, and *Sitobion*, INLV1 provides the first report on a novel nege-like virus in another aphid genus *Indomegoura*. Our results imply that the actual diversity of nege/nege-like viruses in aphids may still be largely undetermined, and the associations between different aphid species and nege/nege-like viruses will be of great interest in future investigation. More intriguingly, it is necessary to further explore the effects of these nege/nege-like viruses (such as INLV1) on aphid competence, and to evaluate whether they can be used as the biological agents to control aphid-borne plant viruses in the field.

## Supplementary Information

**Additional file 1.** File S1: COI sequence of the aphid (Genus *Indomegoura*) used in this study.

**Additional file 2.** File S2: Full genome sequence of Indomegoura nege-like Virus 1.

**Additional file 3.** Table S1: The BLAST results of INLV1 compare to the NCBI NT and NR database.

**Additional file 4.** Table S2: Primers used in this study.

**Additional file 5.** Table S3: Abbreviations of virus names and GenBank accession numbers used in this study.

**Additional file 6.** Figure S1: Prediction of transmembrane domains (TM) in the ORF3 of INLV1. TM 1:68-87 aa; TM 2:107-126 aa; TM 3:138-157 aa; TM 4:177-196 aa.

**Additional file 7.** Figure S2: Alignment of RdRp amino acid sequences of INLV1, previously reported representative nege/kita-like viruses, and plant viruses in the families Kitaviridae and Virgaviridae. Red boxes indicate the position of the motifs - A: DX(4-5)D, B: GX(2-3)TX(3)N, and C: GDD. Virus names and GenBank accessions numbers are listed in Supplementary Table S3.

## Data Availability

The COI sequence of aphids used in this study was stored in GenBank under the accession number MW533423. The full genome sequence of Indomegoura nege-like virus 1 was deposited in GenBank with the accession number MW285725.

## Abbreviations

ISV: Insect specific virus
INLV1: Indomegoura nege-like virus 1
ORF: Open reading frame
UTR: Untranslated region
RdRP: RNA-dependent RNA polymerase
COI: Cytochrome oxidase subunit 1
RACE: Rapid amplification of cDNA ends
ML: Maximum likelihood
siRNA: Small interfering RNA
