# Supplementary material for: Complete genome sequence of a novel nege-like virus in aphids (genus Indomegoura)
Source: Virol J. 2021 Apr 13;18:76. doi: 10.1186/s12985-021-01552-w (PMC8045340; doi:10.1186/s12985-021-01552-w)
Supplement: Supplementary file 3 — Additional file 3. Table S1: The BLAST results of INLV1 compare to the NCBI NT and NR database. [file 12985_2021_1552_MOESM3_ESM.docx]

**Supplementary Table S1. The BLAST results of INLV1 compare to the NCBI NT and NR database.**

| **Blast program** | **Query sequence** | **Top hit viruses** | **Query coverage (%)** | ***E*-value** | **Identify** | **Accession** |
| --- | --- | --- | --- | --- | --- | --- |
| BlastX | full genome of INLV1 | Hubei virga-like virus 4 | 76% | 0.0 | 59.00% | APG77770.1 |
|  |  | Barley aphid RNA virus 1 | 76% | 0.0 | 58.47% | BBV14745.1 |
|  |  | West Accra virus | 75% | 0.0 | 29.42% | BBN20799.1 |
| BlastN | full genome of INLV1 | Hubei virga-like virus 4 | 16% | 5e-50 | 69.85% | KX883814.1 |
| BlastP search | ORF1 protein of INLV1 | Hubei virga-like virus 4 | 100% | 0.0 | 59.00% | APG77770.1 |
|  |  | Barley aphid RNA virus 1 | 100% | 0.0 | 58.63% | BBV14745.1 |
|  |  | Ngewotan nege virus | 98% | 0.0 | 30.60% | AQM55317.1 |
|  | ORF2 protein of INLV1 | Barley aphid RNA virus 1 | 90% | 3e-117 | 44.68% | BBV14746.1 |
|  |  | Hubei virga-like virus 4 | 76% | 2e-89 | 44.04% | APG77771.1 |
|  |  | Wuhan house centipede virus 1 | 93% | 5e-44 | 29.66% | BBV14742.1 |
|  | ORF3 protein of INLV1 | Barley aphid RNA virus 1 | 98% | 6e-73 | 57.21% | BBV14747.1 |
|  |  | Wuhan house centipede virus 1 | 72% | 8e-49 | 51.32% | YP_009342437.1 |
|  |  | Wuhan insect virus 8 | 82% | 1e-45 | 41.62% | YP_009344996.1 |

Abbreviations: INLV1, Indomegoura nege-like virus 1.
