# Supplementary material for: Complete genome sequence of a novel nege-like virus in aphids (genus Indomegoura)
Source: Virol J. 2021 Apr 13;18:76. doi: 10.1186/s12985-021-01552-w (PMC8045340; doi:10.1186/s12985-021-01552-w)
Supplement: Supplementary file 4 — Additional file 4. Table S2: Primers used in this study. [file 12985_2021_1552_MOESM4_ESM.docx]

**Supplementary Table S2. Primers used in this study.**

| **Primer** | **Sequence (5' to 3')** | **Purpose** |
| --- | --- | --- |
| Long primer | CTAATACGACTCACTATAGGGCAAGCAGTGGTATCAACGCAGAGT | Amplification of 5′/3′ RACE fragment |
| Short primer | CTAATACGACTCACTATAGGGC |  |
| 5′-RACE GSP | CAACAACATCGTACCCGTTGACTGGCACTG | Amplification of 5′ RACE fragment of INLV1 |
| 3′-RACE GSP | CGTTCGCTCGCCATTACCTAAACAGAATGC | Amplification of 3′ RACE fragment of INLV1 |
| INLV1-F | AGATTACACCGTCGTCAGTGCCAGT | Amplification of INLV1 viral genome |
| INLV1-R | TGCAGAGAAATCATGATTAATAATA |  |

Abbreviations: GSP, Gene Specific Primer; INLV1, Indomegoura nege-like virus 1.
