# Supplementary material for: Complete genome sequence of a novel nege-like virus in aphids (genus Indomegoura)
Source: Virol J. 2021 Apr 13;18:76. doi: 10.1186/s12985-021-01552-w (PMC8045340; doi:10.1186/s12985-021-01552-w)
Supplement: Supplementary file 5 — Additional file 5. Table S3: Abbreviations of virus names and GenBank accession numbers used in this study. [file 12985_2021_1552_MOESM5_ESM.docx]

**Supplementary Table S3.Abbreviations of virus names and GenBank accession numbers used in this study.**

| **Abbreviations** | **Complete virus name** | **Accession (genome)** | **Accession (RdRp)** |
| --- | --- | --- | --- |
| BARV-1 | Barley aphid RNA virus 1 | LC516835 | BBV14745 |
| HVLV-4 | Hubei virga-like virus 4 | KX883814 | APG77770 |
| WHCV-1 | Wuhan house centipede virus 1 | NC_033469 | YP_009342435 |
| WhIV-8 | Wuhan insect virus 8 | NC_033707 | YP_009344994 |
| MaV | Manglie virus | MH807827 | QBR99594 |
| LORV | Loreto virus | JQ675610 | AFI24687 |
| BCPV | Big Cypress virus | NC_034152 | YP_009351821 |
| BARV-2 | Barley aphid RNA virus 2 | LC516836 | BBV14748 |
| BARV-3 | Barley aphid RNA virus 3 | LC516837 | BBV14751 |
| BARV-4 | Barley aphid RNA virus 4 | LC516838 | BBV14754 |
| AGV-3 | Aphis glycines virus 3 | KX604242 | ASH89118 |
| HGSV-2 | Hibiscus green spot virus 2 | NC_016141 | YP_004928118 |
| CiLV-C | Citrus leprosis virus C | NC_008169 | YP_654538 |
| CiLV-C2 | Citrus leprosis virus C2 | NC_038848 | YP_009509062 |
| HiCV | Hibiscus-infecting cilevirus | MG253805 | ATW76030 |
| SVD1 | Sandewavirus dungfly1 | MT344121 | QOJ43136 |
| TANAV | Tanay virus | MG551504 | AYU75416 |
| BUSV | Bustos virus | LC103139 | BAU71147 |
| NEGV | Negev virus | KX518776 | AQM55314 |
| WAV | West Accra virus | LC496489 | BBN20799 |
| CNLV2 | Culex negev-like virus 2 | NC_035123 | YP_009388582.1 |
| CsV | Castlerea virus | KX903295 | AQZ55390 |
| YKV | Ying Kou virus | NC_040636 | YP_009552739 |
| NVD1 | Nelorpivirus dungfly1 | MT344120 | QOJ43133 |
| SANV | Santana virus | JQ675606 | AFI24675 |
| AHNLV | Andrena haemorrhoa nege-like virus | NC_040813 | YP_009553581 |
| PIUV | Piura virus | NC_034155.1 | AQM55373 |
| DaV1 | Daeseongdong virus 1 | NC_028487.1 | YP_009182191 |
| TMV | Tobacco mosaic virus | NC_001367.1 | NP_597746 |
| CGMMV | Cucumber green mottle mosaic virus | NC_001801.1 | NP_044577 |
