# Supplementary figures and images for: Complete genome sequence of a novel nege-like virus in aphids (genus Indomegoura)
Source: Virol J. 2021 Apr 13;18:76. doi: 10.1186/s12985-021-01552-w (PMC8045340; doi:10.1186/s12985-021-01552-w)

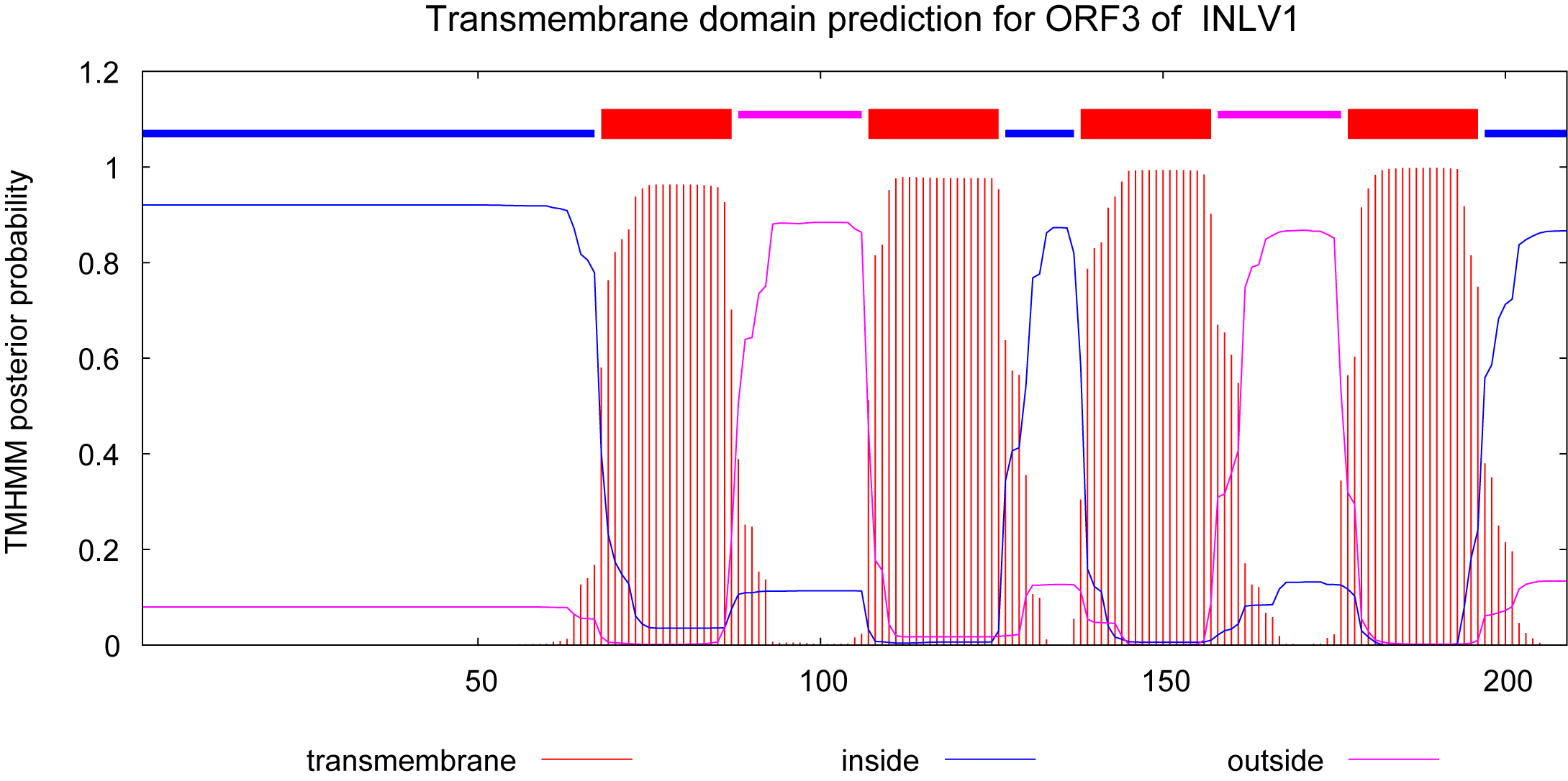

Supplement: Supplementary file 6 — Additional file 6. Figure S1: Prediction of transmembrane domains (TM) in the ORF3 of INLV1. TM 1:68-87 aa; TM 2:107-126 aa; TM 3:138-157 aa; TM 4:177-196 aa. [file 12985_2021_1552_MOESM6_ESM.tif]

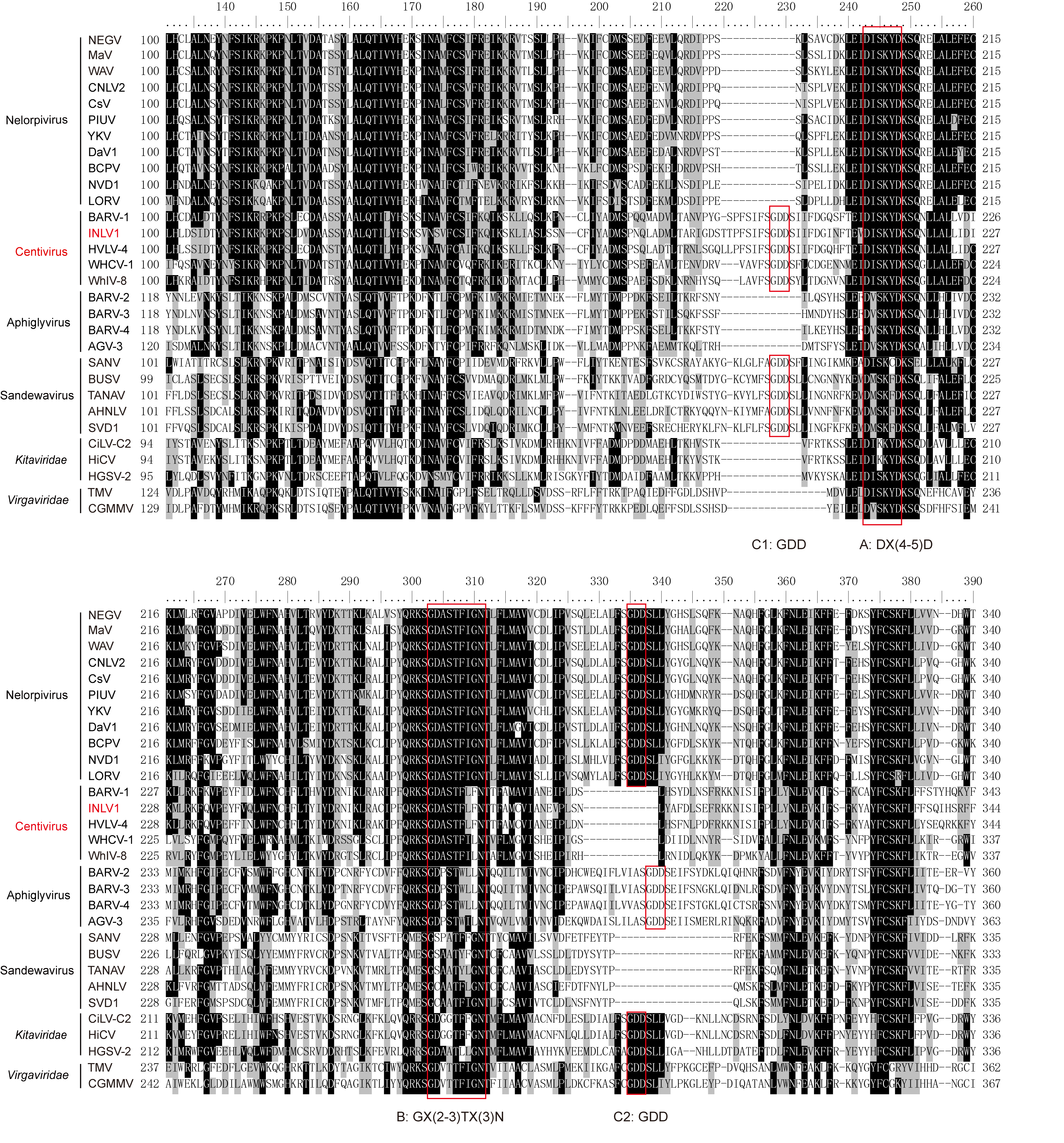

Supplement: Supplementary file 7 — Additional file 7. Figure S2: Alignment of RdRp amino acid sequences of INLV1, previously reported representative nege/kita-like viruses, and plant viruses in the families Kitaviridae and Virgaviridae. Red boxes indicate the position of the motifs - A: DX(4-5)D, B: GX(2-3)TX(3)N, and C: GDD. Virus names and GenBank accessions numbers are listed in Supplementary Table S3. [file 12985_2021_1552_MOESM7_ESM.tif]
